# A Microwave-Based Microfluidic Cell Detecting Biosensor for Biological Quantification Using the Metallic Nanowire-Filled Membrane Technology

**DOI:** 10.3390/s22093265

**Published:** 2022-04-24

**Authors:** Atena Amanati Shahri, Amir Hossein Omidvar, Gustavo Pamplona Rehder, Ariana Lacorte Caniato Serrano

**Affiliations:** Escola Politécnica, Universidade de São Paulo, São Paulo 05508-010, Brazil; a.omidvar@usp.br (A.H.O.); gprehder@usp.br (G.P.R.); aserrano@usp.br (A.L.C.S.)

**Keywords:** microwave sensors, microfluidics, biosensors, change in permittivity, single-cell measurement

## Abstract

A label-free, sensitive, miniaturized sensing device was developed for detecting living cells in their flow stream. The outstanding performance of this biosensor in distinguishing living cells in cell suspension was achieved by integrating microstrip stub resonator above a microfluidic structure using the metallic nanowire-filled membrane technology. The cell suspension flows in a microfluidic channel placed between the tip of the stub resonator and its ground plane as the substrate to take advantage of the uniform and concentrated field distribution. We studied the changes in relative permittivity due to the presence of a single living cell in the phase of the transmitted signal (S_21_). An average variation of as much as 22.85 ± 1.65° at ~11.1 GHz is observed for the living cell sensing using this optimized device. This biosensor could detect rapid flowing cells in their biological medium in real-time and hence, can be used as an early diagnosis and monitoring tool for diseases.

## 1. Introduction

Observing and investigating cells is a common approach used in multiple medical applications such as pharmaceutical, biotechnology, and other clinical/medical research activities. Characterizing cells promptly is a crucial element of effective medical diagnosis and treatment. Assessment of cell counting is an essential step in the characterization of cells. Currently, cell counting is being performed either manually or automatically. Manual cell counting suffers from various shortcomings such as time-consuming preparations, accuracy due to subjectivity of counts, and device misuse. Automated cell counters offer more reliable and reproducible results by partially eliminating human errors from the workflow.

Conventionally, optical detection systems are utilized for cell analysis due to their high efficiency and specificity on account of fluorochromes and antibodies used for attachment in the cells. Yet, these methods are considered invasive for observing cells due to labeling techniques to analyze intracellular structures. These labels and markers need time to interact with the cells; therefore, real-time monitoring is not an option. At the same time, cells must first be fixed, stained, and then analyzed. The procedure is time-consuming and also destructive for the cells. Moreover, costly equipment for further analysis and information extraction poses unique challenges. Thus the development of techniques that would be non-invasive or label-free has the potential to improve biological discovery significantly.

Lately, biosensing has experienced enormous growth by deploying microwave technologies for healthcare applications. The possibility of detecting the presence of microorganisms at the cell level and measuring the property of a single cell can pave the way for novel medical applications. Therefore, these biosensors are beneficial in developing point-of-care testing, from monitoring treatment to disease progression. 

The use of electromagnetic (EM) waves in the frequency range of GHz to THz in biosensors as the detection technique for the characterization of biological suspensions and biomolecules was reported successfully [1]. Microwave biosensing has valuable advantages, such as offering non-invasive, rapid, and label-free detection, and has the potential to be implemented commercially. Several microwave measurement methods and techniques for characterizing material properties at microwave frequency have been developed and utilized with high accuracy [2,3,4,5,6,7,8]. Amongst all, planar transmission line techniques have been widely used to determine the dielectric properties of cells.

Different frequency ranges and design methods have been proposed in the literature to study the concentration, population, and viability of cells. There was a significant focus on developing coplanar waveguide-based (CPW) biosensors in the broad frequency range up to 40 GHz to study changes in the relative permittivity of cell suspensions for biological quantification. Amicrofluidic channel was placed on top of a coplanar waveguide (CPW) structure to extract the permittivity of human umbilical vein endothelial cell concentration when the sensor was loaded with biological medium compared to when it was loaded with high concentration cell suspension (1.25 × 10^8^ cells/mL) [9]. Later, the same sensor was utilized to determine the dielectric signatures of various concentrations of tumorous cells (1, 10, 100 million cells/mL) [10]. However, highly concentrated cell suspensions were used to reduce the undesired effect of the lossy aqueous suspending medium for better sensitivity. The center of the signal line in CPW-based biosensor was etched to create a microchamber on the biosensor surface to detect various concentrations of cancer cells (HepG2) based on their dielectric properties [11]. Still, the biomedical protocol needed a long time of preparation. A capacitive gap was designed at the center of the coplanar line to detect a single living B lymphoma cell in its culture medium where the cell was mechanically trapped [12]. The maximum contrast was obtained around 5 GHz. The capacitive difference between living and affected monocyte cells with electroporation at the single-cell level, and the response of two different cells under chemical stimulus were investigated with the same sensor in [13,14]. However, In CPW-based biosensors, due to the field distribution of the coplanar waveguide configuration, around half of the electrical fields are located in the fluidic channel, whereas the other half passes through the substrate.

Microwave biosensors have also been utilized to study permittivity changes through components such as resonators and microstrip structures with high accuracy. They have advantages, such as low cost, easy fabrication, and being small and light [15]. Resonant-based methods are good examples of sensitive narrowband detection of biomarkers, DNA, the concentration of biomolecules and beads in liquid solutions, and biological cells in several applications [16,17,18]. Resonant-based detection techniques can distinguish the changes in relative permittivity of the material under test (MUT), observing shifts in the frequency of S-parameters at resonance frequencies. Meandered inductors coupled to inter-digital capacitors were proposed to identify different human cell types [19,20], and to characterize the degree of aggressiveness of malignant cells based on their dielectric permittivity [21]. To avoid the effect of the biological medium, the cells were either in a particular support media based on a polymer matrix (ficoll) or directly deposited and grown on the biosensor surface. The number and position of the cells were uncontrolled due to the lack of a microfluidic channel, and the cells were not in their living medium. Therefore, a limited number of cells can be characterized, and yet the prolonged biochemical preparation and confirmation of the position of the cells in the sensing area were inevitable. A substrate-integrated waveguide (SIW) cavity resonator was used to analyze the fibroblast (FB) cells in a cup-shaped container [22]. A microwave hairpin resonator was designed to obtain B16F10 melanoma cell characteristics in a liquid medium [23]. A coupled-resonator biosensor composed of a double split-ring resonator (DSRR) coupled with a cylindrical dielectric resonator (DR) for measuring single flowing cells in an aqueous environment was proposed [24].

In the reviewed literature, several challenges were revealed, including the integration of microfluidic systems, and using complicated, slow, time-consuming, and expensive preparation work. These cause some drawbacks, such as the high cost of fabrication to improve the selectivity and sensitivity of the biosensors. However, new biosensing technology should represent a promising solution to enhance biological and medical applications as a rapid diagnostic tool. It should be simple, miniaturized, and cost-effective to serve as a point of care device while providing real-time measurement. Yet the sensitivity of the biosensor is the main limitation. For this purpose, the size of the lines should be fine-tuned to match the cell size to eliminate the effect of the surrounding liquid or area. In addition, measuring the rapid-flowing cell in the microfluidic channel was another difficulty, and mechanical elements were used to trap the cell and increase selectivity. This will add more complexity to the fabrication process hence more cost. Moreover, in those articles, the fluidic channels or chambers are integrated on the top surface of the transmission line, which results in less sensitive responses considering the electric field distribution in the MUT. 

The device proposed here integrates a microfluidic structure underneath a parallel stub resonator based on microstrip transmission line for an accurate dielectric measurement where the liquid under test was located in the place of the maximum uniform electric field within the sample. Hence a single flowing cell in its biological medium was detected without being mechanically or electrically trapped, from the phase shift of the transmitted signal as a result of changes in the relative permittivity caused by the cell presence. This microwave-based microfluidic biosensor was fabricated using the metallic nanowire-filled membrane technology made of porous alumina [25], which is biocompatible, being an appropriate choice for both microfluidics and microwave applications.

## 2. Materials and Methods

### 2.1. Sensor Design

**Figure 1:**
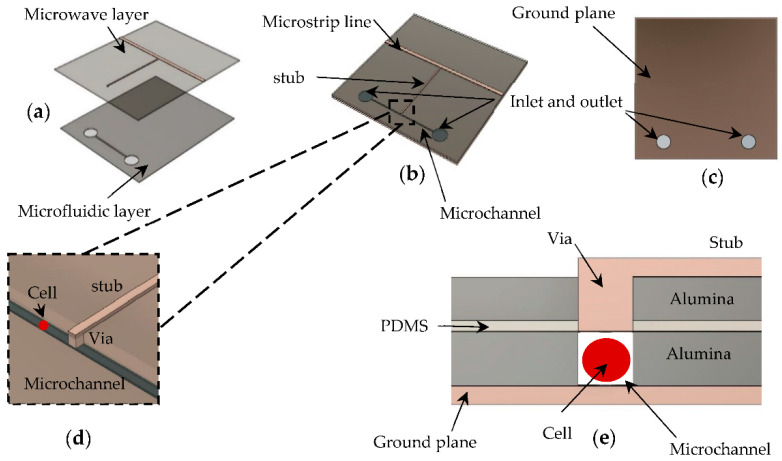
The designed microwave-based microfluidic biosensor: (**a**) Expanded view of the two layers composing the integrated biosensor; (**b**) View of the assembled biosensor; (**c**) Bottom view of the assembled biosensor showing the inlet and outlet; (**d**) Close up of the stub tip above the microchannel; (**e**) Cross-sectional view of the microfluidic biosensor when the cell suspension flows in.

Figure 1a shows that the proposed biosensor integrates two layers. The one at the bottom is a microfluidic layer where a microchannel is placed under a microwave layer. The microwave layer is formed by a microstrip transmission line and a parallel stub resonator with a via on its tip. This layer is used to confine and seal the microchannel filled with cell suspensions to be characterized. The microfluidic layer is aligned with the top layer, as shown in Figure 1b. The ground plane at the back of the microfluidic layer can be seen in Figure 1c. The via extends the tip of the stub resonator to precisely above the channel in which the cell suspension flows orthogonally to the stub, as shown in Figure 1d; therefore, the cell suspension is the only medium between the tip of the stub and the ground plane. A cross-sectional view of the microfluidic biosensor when the cell suspension flows in is shown in Figure 1e. 

Here, porous alumina substrate from InRedox (relative permittivity of 6.7 and tan*δ* of 0.025) was chosen due to its outstanding biocompatibility. It is optically transparent. This feature facilitates the visual detection and direct imaging of cells in the microfluidic channel using optical microscopy. Furthermore, it is simple to fabricate a microchannel with a width small enough to allow passing of only one cell at a time and a small-diameter metal via through it. In this case, the via composed of metallic nanowires stretches out the stub tip inside the membrane, confining the electric field between the ground plane and the top of the metallic nanowires inside the sensing region. This means that the electric field only passes through the microchannel and not through the porous alumina substrate, and enhances the sensibility of the sensor. 

**Figure 2:**
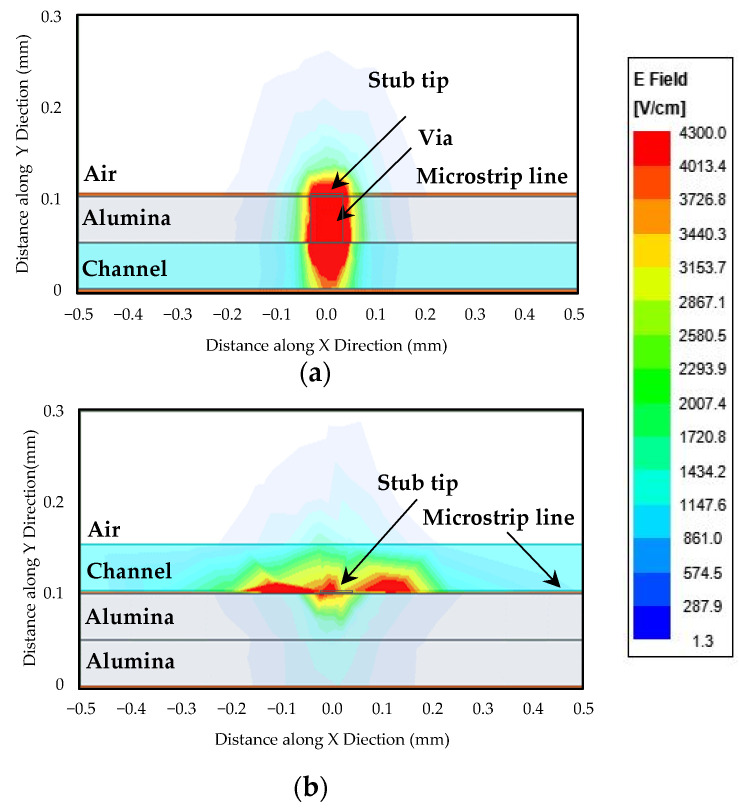
Position of the microchannel and the electric field distribution in: (**a**) the microfluidic biosensor proposed in this work; (**b**) sensor with the microfluidic channel above the transmission line.

Figure 2 illustrates the importance of the position of the channel and gives a better understanding of how the electric field distribution was affected dramatically by the material surrounding the resonator in two different configurations. In the structure shown in Figure 2a, the 50 μm thick microfluidic channel was located inside the 50 μm thick alumina substrate at the bottom. The 50 μm width via extending the stub tip to the microchannel inside 50 μm thick alumina results in the maximum uniform electric field within the sample; therefore, higher sensitivity in detecting living cells is obtained due to the intense interaction of the field with the biological samples. 

In Figure 2b, the same two 50 μm thick alumina membranes were considered the dielectric substrate between the microstrip and the ground plane. The 50 μm thick microchannel was now placed on top of the stub. The microchannel was filled with distilled water (DI water) (relative permittivity (ε_r_ ) ~80) in these figures. Ansys Electronics Desktop was used as the 3D EM simulator to calculate these quantities. 

These figures do not provide a direct comparison between our proposed design and the works in the literature since those works used different substrate materials (glass commonly), and the majority of them utilized PDMS covers or channels aside from different methods of data extraction. The main focus here was to demonstrate the effect of the microchannel position relative to the transmission line. It can be easily seen that the electric field is much more confined in the sensing region inside the microchannel in our proposed device as opposed to when the microchannel is placed on top of the transmission line. Therefore, for this specific application that detecting a microorganism at the cell level (microscale) is the aim of the study, our proposed sensor provides a much greater intensity of electric field hence more interaction with cells. The sensing area exactly under the stub tip matches the cell size and is the most sensitive area to permittivity changes.

**Figure 3:**
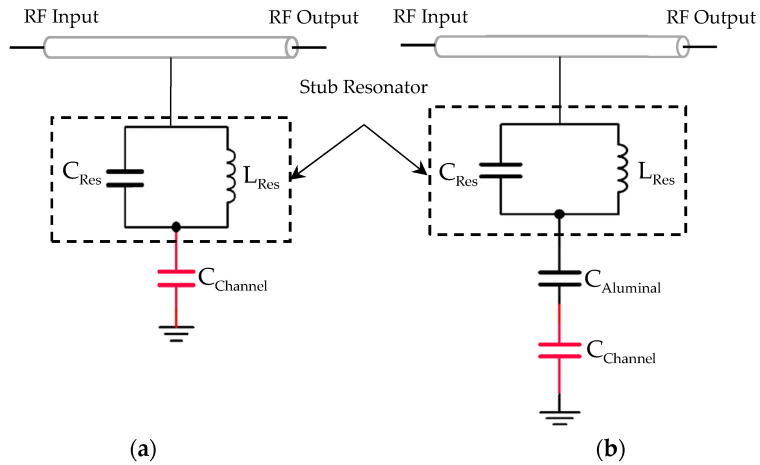
Electrical equivalent model of (**a**) our proposed biosensor with via on the tip of stub; (**b**) sensor without via.

An equivalent circuit model of our proposed sensor is shown in Figure 3a. The circuit is a transmission line with a microwave resonator (the stub) that can be modeled by an inductance (L_Res_) and a capacitance (C_Res_) followed by a capacitance at its tip to the ground, related to the microchannel, shown as C_Channel_. Changing this capacitance, the electrical length of the stub is altered; thus, the resonance changes. In fact, when a cell with different permittivity to the surrounding medium enters the sensing region under the stub, it changes this capacitance (C_Channel_). This translates to a change in the transmitted microwave signal phase.

If the alumina substrate was placed in between the tip of the resonator and the microchannel, without the via, the changes in the total capacitance would be highly attenuated as the cell and the medium have relative permittivity (ε_r_) well higher than the porous alumina due to their water content. With the capacitance of the alumina (C_Alumina_) and the cell suspension (C_Channel_) in series, as illustrated in the equivalent model in Figure 3b, the material with lower relative permittivity, hence lower capacitance, attributed to the total capacitance. This means that, in the presence of an alumina layer (ε_r_ ~7), even a big difference in the relative permittivity of the material in the microchannel from liquid (ε_r_ ~80) to cell (ε_r_ ~40) would not affect the total capacitance significantly.

**Figure 4:**
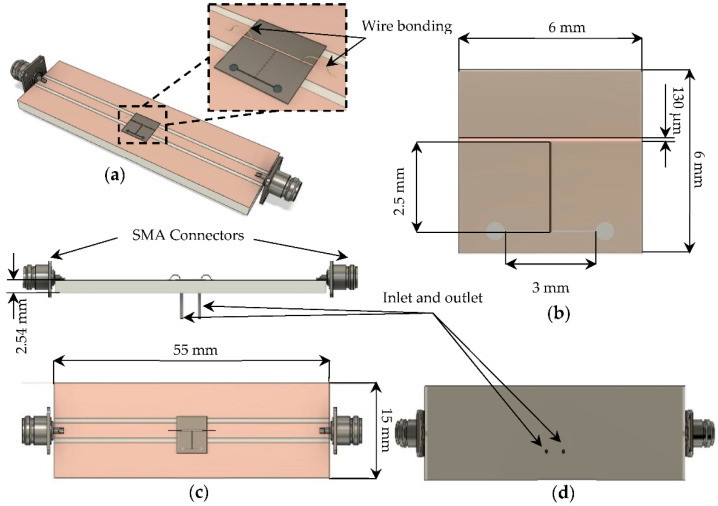
The model of the fully assembled biosensor to the carrier: (**a**) Close up of the microfluidic biosensor wire bonded to the carrier; (**b**) Top view of the microfluidic biosensor showing its dimensions; (**c**) Top view and lateral view of the fully assembled biosensor to the carrier showing the dimensions of the carrier; (**d**) Bottom view of the carrier showing the structure of liquid injection accesses.

The microfluidic biosensor, shown in Figure 4, was fabricated with a length of 6 mm and a width of 6 mm, where we have constructed a 3 mm long and 50 μm width microchannel in the bottom layer. On the top alumina layer, we have a 50 Ω microstrip transmission line with a length of 6 mm and width of 130 μm, and a quarter wavelength stub with a length of 2.5 mm and width of 50 μm. A 50 μm × 50 μm square-shaped via was placed on the tip of the stub inside the membrane. Finally, a carrier was fabricated to provide mechanical stability to the structure, especially regarding the liquid injection accesses (inlet and outlet). Using the carrier, the sensor could be measured by SMA connectors, and the use of expensive probes was avoided, which could be damaged if there was any leak. The carrier provided 50 Ω coplanar waveguide (CPW) access (input and output of the device), and was fabricated on a Rogers TMM 6 microwave laminate (relative permittivity of 6 and tan*δ* of 0.0023). The inlet and outlet were drilled on the carrier. The TMM6 substrate was 15 mm wide, 55 mm long, and 2.54 mm thick.

### 2.2. Sensor Fabrication

**Figure 5:**
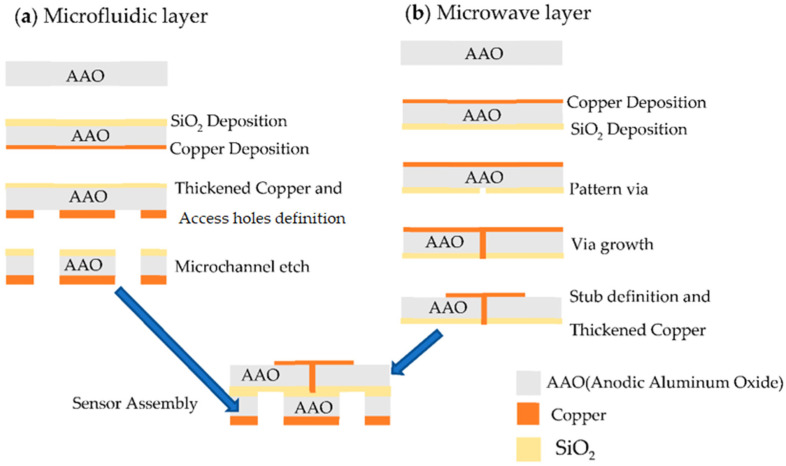
Fabrication steps of the microfluidic and microwave layers for our proposed biosensor.

The microfluidic biosensor was fabricated using standard microfabrication techniques and divided into two main steps: (1) fabrication of the microfluidic and microwave layers illustrated in Figure 5, and (2) assembly of the two layers to form the microchannel and integration onto the carrier substrate. For the microfluidic layer, first, a 20 nm thick copper seed layer was deposited by RF magnetron sputtering. This layer was then patterned with the ground plane geometry by direct laser lithography. The patterned copper was thickened to 3 μm by electroplating. On the other side, silicon dioxide (SiO_2_) was deposited by sputtering to be used as a mask for the channel etching. The microchannel geometry was patterned, and the microchannel was etched with a NaOH (50%) solution at 50 °C. 

The same copper seed layer and SiO_2_ layer used in the microfluidic layer were deposited on the top and bottom surfaces of the microwave layer. The SiO_2_ was patterned with the via geometry, and copper was grown through the porous aluminum to form a via, as described in [25]. The copper seed layer was patterned, and the copper was thickened to 3 µm to form the microstrip line and the stub. 

The two layers were assembled under a microscope using PDMS to seal the channel. The carrier substrate was fabricated using a milling machine, and the microfluidic biosensor was attached to it using electrically conductive silver epoxy. Ultimately, the microstrip line on the biosensor was wire bonded to the CPW signal line on the carrier connecting the RF input to the output. 

### 2.3. Measurement Setup

The performance of our proposed microfluidic biosensor has been characterized in terms of network S-parameters using a performance network analyzer (PNA) (N5227B, Keysight, Santa Rosa, CA, USA) in two ports of configuration after the SOLT calibration process up to 20 GHz. The measurement resolution of 0.1 dB, 1 Hz, and 0.5° in terms of magnitude, frequency, and phase, respectively. The device under test (DUT) is connected to PNA ports via coaxial cables. All experiments were carried out under normal laboratory environment conditions.

**Figure 6:**
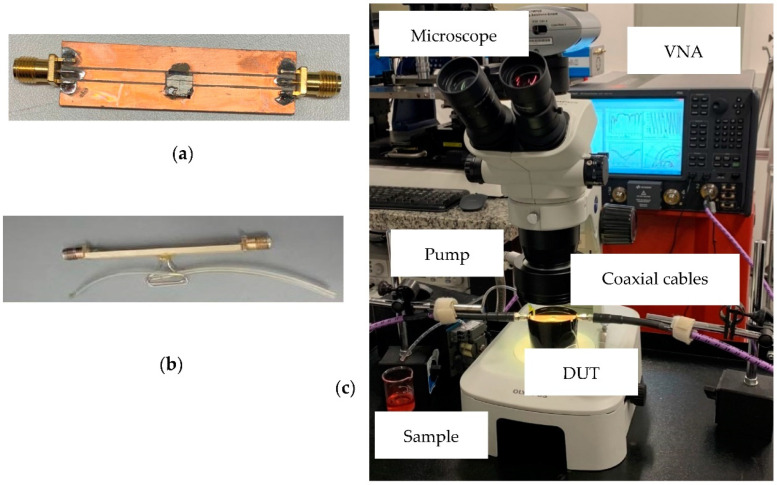
Photograph of (**a**) Top view of fabricated sensor; (**b**) Lateral view with liquid injection accesses and tubing; (**c**) Experimental set up with equipment.

We used the S_21_ scattering parameter of our device to characterize the cell suspensions when the liquid is stabilized in the sensing area. Figure 6a,b shows the fabricated sensor with injection accesses, and Figure 6c shows the complete experimental setup for the measurement: VNA, DUT, coaxial probes, pump, microscope, and the cell suspension.

In the process, each time, first, the response of the sensor with an empty microchannel was measured, then the microchannel was filled with the biological medium continuously as a reference to check the reproducibility and repeatability of the study, and finally, filled with cell suspensions. Furthermore, various duplicated sensors from the same fabrication process were elaborated to validate the performance of the biosensor at several attempts. The liquid flow was controlled using a pump, and the microfluidic channel was monitored with a microscope simultaneously. Furthermore, a high-speed camera recorded the sensing area continuously while the magnitude and phase of S_21_ were measured as soon as the liquid flowed in the microchannel. A similar set of experiments with the same procedure was carried out for the cell suspension in different concentrations.

### 2.4. Electromagnetic Analysis

**Figure 7:**
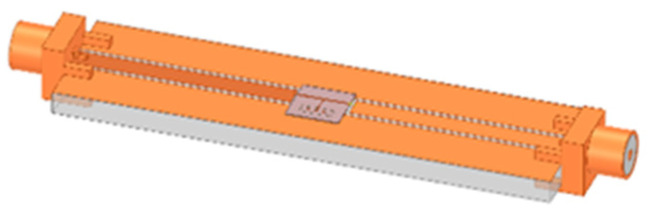
HFSS model of our proposed microwave-based microfluidic sensing device.

**Figure 8:**
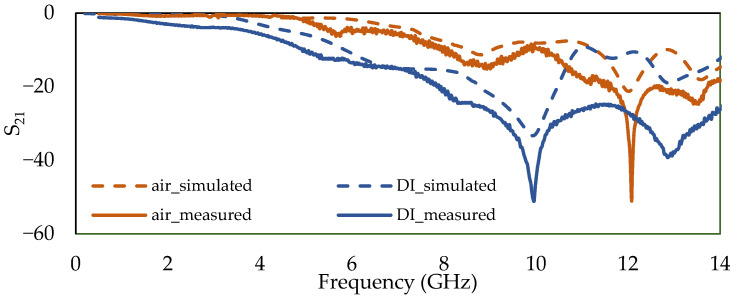
Simulated vs. measured frequency response of the insertion loss (S_21_) for empty microchannel and filled with DI water.

Ansys Electronics Desktop was used as the 3D EM simulator to analyze the performance of the biosensor in terms of magnitude, phase, and frequency responses using the modeled structure in Figure 7. As proof of concept, a set of simulations were carried out in which the microchannel was first considered empty, and then was filled with DI water with relative permittivity of 80. The simulated (dashed orange) and measured (straight orange) frequency response of the empty channel (filled with air), and the measured frequency response of the microchannel filled with DI water (straight blue) compared to the simulation (dashed blue), up to 14 GHz, are presented in Figure 8. The agreement between simulations and measurement is good for both experiments, validating the simulation of the sensor’s behavior in Ansys. The differences between the curves are mainly due to the wire bonding connecting the sensor to the carrier. 

## 3. Results

### 3.1. Cells’ Description and Preparation

**Figure 9:**
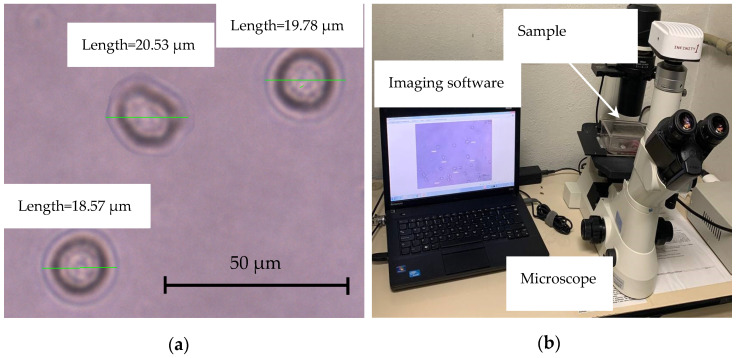
(**a**) Close-up of the cell size and shape measurements using optical microscope images treated by image analysis software; (**b**) Complete setup for cell size measurement.

To evaluate the sensing capability of the proposed biosensor, C1498 cells with a typical diameter close to 20 μm were studied. Their round shape facilitates the visualization process. The C1498 cells (ATCC^®^ TIB-49) were cultivated in a 75 cm^2^ tissue culture flask with Dulbecco’s Modified Eagle Medium (DMEM) supplemented with 10% Fetal Bovine Serum. The flask was incubated at 37 °C with 5% CO_2_, and the medium was changed every three days; then, all the contents of a flask (cell and medium) were transferred in a 50 mL tube and centrifuged at 125× *g* for 10 min. After discarding the supernatant, the pellet of cells was re-suspended with fresh DMEM medium at the dilution ratio of ~2 × 10^5^ cells/mL. Cell suspensions were prepared under ethical standards. Optical methods and image analysis software were used to measure the diameter of cells, as shown in the setup in Figure 9. The cells were placed in their respective fresh culture medium before the measurements.

### 3.2. Real-Time Cell Measurements

**Figure 10:**
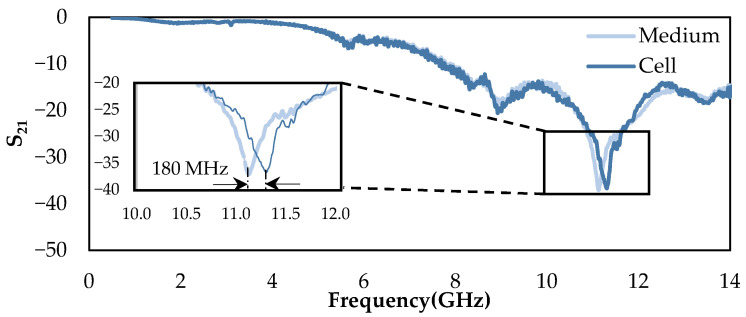
Measured frequency response of the insertion loss (S_21_) for the medium compared to the single-cell presence under the stub in the sensing region.

To verify the performance of the biosensor, the real-time measurement was divided into two main steps. First, the sensor was loaded only with the biological medium (without cells) to study where the structure presents an absorption peak. Then cell suspension flowed in the microchannel, which was constantly monitored. The moment a single cell traveled through the microfluidic channel, the flow rate of the cell suspension was reduced to ensure the presence of the cell strictly under the stub tip in the sensing region. Then the frequency response of the biosensor was recorded. Figure 10 shows the frequency response of the medium compared to the instance that a cell is under the stub tip. The presence of a single cell affects the magnitude and frequency of the absorption peak; a shift of 180 MHz in the absorption peak can be observed, as shown in the inset of Figure 10.

In the second step, to detect a single living cell, we conduct real-time measurements, with an alternative approach, by operating the VNA in continuous wave (CW) mode at the absorption peak (~11.1 GHz) with a power level of 0 dBm, and an IFBW of 3 kHz to study the phase of S_21_ scattering parameter. The CW mode provides significant control to capture and record the changes due to the speed of the cells proceeding through the microchannel. 

**Figure 11:**
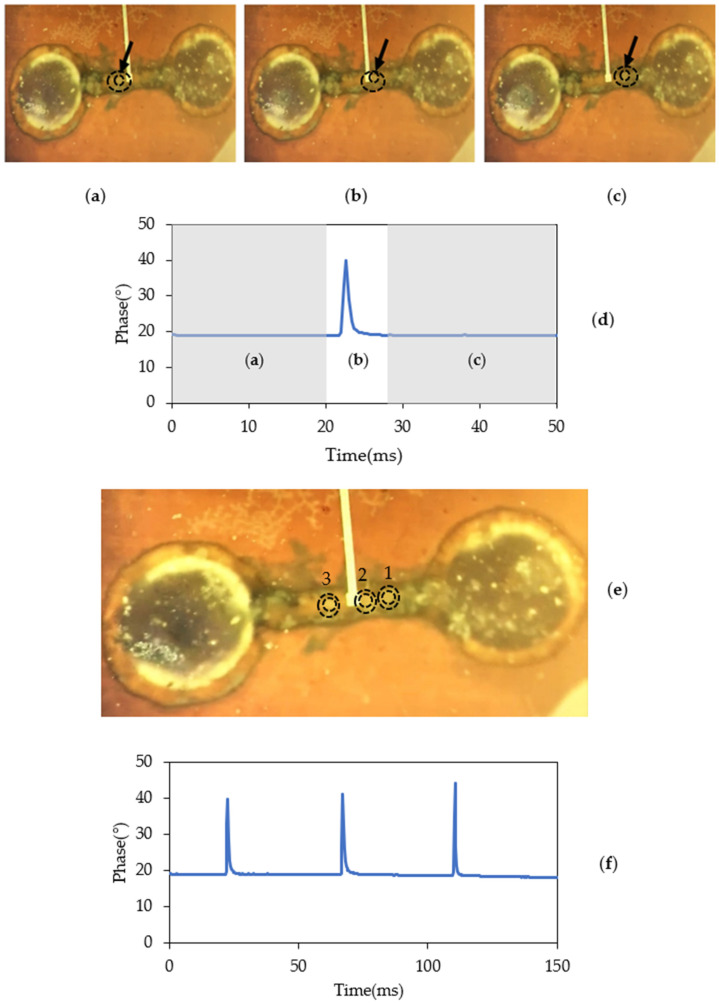
Real-time measurements of a single C1498 cell: (**a**–**c**) are optical microscope images captured by the high-speed camera displaying a C1498 cell (indicated by black circles and arrows) entering the microchannel from the inlet (left), crossing the sensing region, and moving forward toward the outlet (right), respectively; (**d**) displays the real-time measurement of phase data of a single cell moving forward in a channel for each time point from (**a**–**c**) in 50 ms; (**e**) displays an image of three cells (circled in black) during the same pass through the microfluidic channel; (**f**) shows the phase data from the biosensor showing a total of 3 consecutive cell passes.

Figure 11 demonstrates pictures taken during the real-time measurements of the cells. Figure 11a–c are optical microscope images captured by the high-speed camera displaying a C1498 cell (indicated by black circles and arrows) entering the microchannel, crossing the sensing region, and moving forward toward the outlet, respectively. We confirmed that a single cell passed through the channel by constantly monitoring the microchannel. The same cell was moved backward and forward through the region five times to verify the responses. The real-time phase data for the transmitted signal for each time point shown in Figure 11a–c were extracted. The shift in the transmitted signal phase was observed in Figure 11d. A peak confirmed the presence of the cell in the sensing region underneath the stub tip in the microfluidic channel. Figure 11e displays an image of three cells during the same pass through the microfluidic channel. The cells are circled in black, traveling from left to right in the microchannel. Figure 11f illustrates the real-time phase data for three consecutive cell passes. Each peak value in the phase of S_21_ presented in this figure corresponds to the presence of a single cell. An average of 22.85 ± 1.65° shift in the phase of the transmitted signal, in the presence of the cell in the medium, were measured. To avoid the incident where multiple cells passed the sensing area, the direction of the cell suspension was changed various times randomly while cells moved forward and backward. The obtained results eliminated the possibility of overlapping cells’ measurement, which was not the case in the present work, and gave us excellent reproducibility.

## 4. Discussion

Table 1 summarizes the label-free microwave-based sensors for the dielectric characterization of cells using planar transmission lines techniques stated in the literature and that of our proposed sensor. The studied frequency range, design methods, and microwave components are also presented in this table.

The studies using adherent cells provide less sensitivity than the sensing device proposed in this work. Moreover, the process is aggressive to the cell and may modify its properties or destroy it. One way to improve this methodology is to use a microfluidic channel with flowing cells. However, some works use mechanical components to fix the position of the cell [23]. This adds complexity to the fabrication process. In this work, we detect the continuous flowing cells, and high sensitivity was achieved in terms of phase due to its particular design. By placing the tip of the microstrip stub resonators above the microfluidic structure through a via, the cell suspension is set to be the mediumbetween the tip of the stub resonator and its ground plane. Therefore, it can be argued that the obtained response of flowing single cells at microwave frequencies in the sensing region is due to the different relative permittivity of the cell with respect to its biological medium crossing the sensing region. Even if it was not our goal in this work, our proposed sensor has a higher sensitivity in terms of insertion loss than the resonator in [24].

This study presented an integrated microwave-based microfluidic biosensor for real-time detecting of flowing cells for cell quantification. The microfluidic channel was integrated into stub resonators using the metallic nanowire-filled membrane technology. The device proposed in the present work distinguished cells measuring the phase shift of the transmitted electromagnetic wave. A variation of as much as 22.85 ± 1.65° degree was observed at ~11.1 GHz. It was demonstrated that the change in the transmitted signal phase is correlated to cells passing through the sensing region. The architecture of this sensor makes it possible to fabricate the microchannel in between the stub and the ground. Hence, the interaction between the sample and the electric field was stronger, leading to a more effective parameter extraction. This biosensor successfully detects cells in their biological medium in real-time hence, it can be used as an early diagnosis and monitoring tool for diseases.

Now that the presence of a cell with respect to the medium over time was detected, this device can be subject to a dramatic improvement. The next step could be to conduct size measurements and cell differentiation by effectively controlling the flow of the cell using an appropriate pump to provide desired pressure and flow of the cell suspension in the channel. Due to their slower velocity, the bigger cells require more time to pass through the sensing region. This transit time can be measured and correlated to the size of the cell. It also qualifies the cell discrimination based on their size.

## Figures and Tables

**Table 1:** Comparison with the State-of-the-art label-free microwave-based sensors.

Ref.	Microwave Topology	Position of the Microfluidic Channel to TL	Flowing or Adherent Cell	Sensing Parameter	Studied Frequency (GHz)
[20]	Interdigitated comb capacitor	-	Adherent	|S_21_|,|S_11_| f_r_	14.7
[12]	CPW	Top	Flowing	|S_21_|,ε_r_	1–40
[21]	Interdigitated comb capacitor	-	Adherent	f_r,_ ε_r_	5–14
[23]	Microstrip resonator	Top	Flowing, mechanically trapped	|S_11_|,|S_21_|	2.17
[24]	Microstrip resonator/dielectric resonator	Top	Flowing	|S_21_|	9.8
**This Work**	**Stub resonator/nanowire- filled technology**	**Under**	**Flowing**	**fr, ** **phase**	**11.1**
